# Scaffolding protein Homer1a protects against NMDA-induced neuronal injury

**DOI:** 10.1038/cddis.2015.216

**Published:** 2015-08-06

**Authors:** Y Wang, W Rao, C Zhang, C Zhang, M-d Liu, F Han, L-b Yao, H Han, P Luo, N Su, Z Fei

**Affiliations:** 1 Department of Neurosurgery, Xijing Hospital, Fourth Military Medical University, Xi'an, P.R. China; 2 Department of Neurosurgery, Wuhan Zhong Xin Hospital, Wuhan, P.R. China; 3 Department of Neurology, Second Artillery General Hospital of PLA, Beijing, P.R. China; 4 Department of Biochemistry and Molecular Biology, State Key Laboratory of Cancer Biology, The Fourth Military Medical University, Xi'an, P.R. China; 5 Department of Medical Genetics and Developmental Biology, State Key Laboratory of Cancer Biology, Fourth Military Medical University, Xi'an, P.R. China

## Abstract

Excessive N-methyl-D-aspartate receptor (NMDAR) activation and the resulting activation of neuronal nitric oxide synthase (nNOS) cause neuronal injury. Homer1b/c facilitates NMDAR-PSD95-nNOS complex interactions, and Homer1a is a negative competitor of Homer1b/c. We report that Homer1a was both upregulated by and protected against NMDA-induced neuronal injury *in vitro* and *in vivo*. The neuroprotective activity of Homer1a was associated with NMDA-induced Ca^2+^ influx, oxidative stress and the resultant downstream signaling activation. Additionally, we found that Homer1a functionally regulated NMDAR channel properties in neurons, but did not regulate recombinant NR1/NR2B receptors in HEK293 cells. Furthermore, we found that Homer1a detached the physical links among NR2B, PSD95 and nNOS and reduced the membrane distribution of NMDAR. NMDA-induced neuronal injury was more severe in Homer1a homozygous knockout mice (KO, Homer1a^−/−^) when compared with NMDA-induced neuronal injury in wild-type mice (WT, Homer1a^+/+^). Additionally, Homer1a overexpression in the cortex of Homer1a^−/−^ mice alleviated NMDA-induced neuronal injury. These findings suggest that Homer1a may be a key neuroprotective endogenous molecule that protects against NMDA-induced neuronal injury by disassembling NR2B-PSD95-nNOS complexes and reducing the membrane distribution of NMDARs.

Glutamate (Glu) acts on glutamate receptors, such as the N-methyl-D-aspartate receptor (NMDAR), and leads to neuronal hyper-excitability and death in a dose-dependent manner.^1^ NMDAR activation induces Ca^2+^ influx and specifically activates neuronal nitric oxide synthase (nNOS) and downstream signaling pathways.^2, 3, 4^ Ca^2+^ influx is involved in glutamate-induced apoptosis caused by the activation of apoptosis-related signaling pathways, mitochondrial dysfunction and ROS induction.^3, 4^ Additionally, nNOS has been reported to contribute to NMDA-induced excitotoxicity.^5, 6^ Considering that direct NMDAR inhibition has not yet demonstrated favorable efficacy in most clinic trails and further considering the remarkable role of nNOS in NMDA-induced neuronal death,^7^ measures that can effectively protect neurons from NMDA-induced neuronal injury are urgently needed and represent a worthwhile research goal.

Homer proteins belong to the postsynaptic density (PSD) family and consist of two major groups: the short-form Homer proteins (Homer1a and Ania3) and the long-form Homer proteins (Homer1b/c, Homer2 and Homer3).^8^ Homer1b/c has a conserved N-terminal Ena/VASP homology 1 domain and binds to group I metabotropic glutamate receptors (mGluRs), inositol triphosphate receptors and Shank family proteins.^9, 10, 11, 12^ Homer1b/c regulates surface receptor expression,^13, 14^ clustering,^15^ transient receptor potential family channels and mGluRs coupled to ion channels.^10, 16, 17, 18, 19^ Additionally, because of its C-terminal coiled-coil (CC) domains, Homer1b/c can self-multimerize, form multiprotein complexes and facilitate signal transduction to downstream pathways. Homer1a, which lacks the CC domain, is believed to compete with constitutive Homer1b/c and disrupt the association of multiple Homer1b/c complexes.

Notably, Homer1b/c can interact with the Glu-induced Ca^2+^ influx pathway by binding to Shank, a NMDAR complex adaptor protein (NMDAR-PSD95-GKAP-Shank-Homer1b/c).^12, 20^ Furthermore, Homer1a also interacts with Shank, NMDA, nNOS and other Homer1b/c target proteins. Homer1a has a negative regulatory role by physically replacing certain target proteins, and is involved in the regulation of a variety of cellular and molecular functions in neurological diseases.^21, 22, 23, 24, 25^ Nevertheless, the mechanisms of action and associations between Homer1a and NMDA-induced neuronal injury have not yet been studied. Here, we aimed to investigate the possible neuroprotective effects of Homer1a and explore the mechanisms underlying Homer1a activity in NMDA-induced neuronal injury.

## Results

### Verification of NMDA-induced neuronal injury *in vivo* and *in vitro*

**Figure 1:**
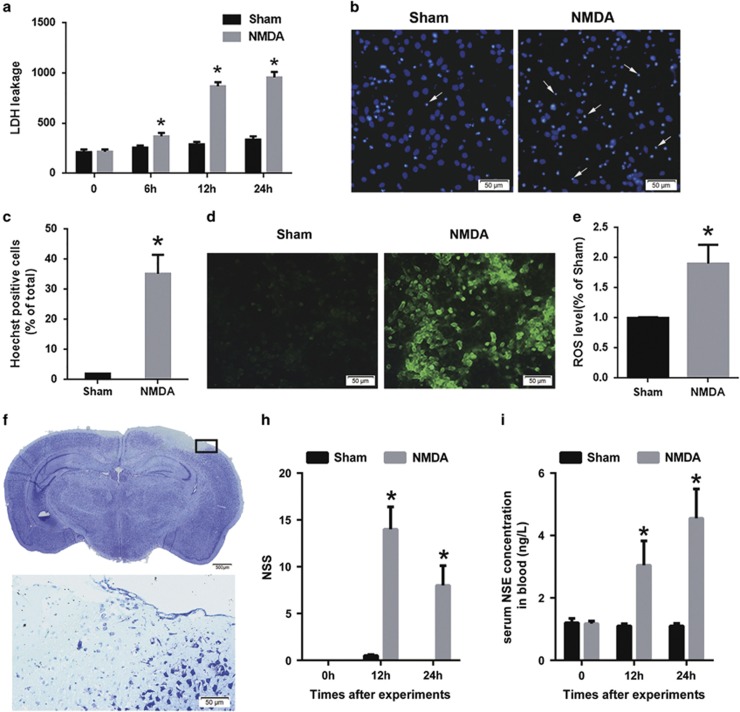
Evaluation of the NMDA injury model *in vitro* and *in vivo*. After NMDA injury in cultured cortical neurons, cytotoxicity was determined by the LDH assay (**a**) and the apoptotic rate was assessed by Hoechst staining (arrows: apoptotic neurons; Scale bar=50 *μ*m; (**b** and **c**)) ROS production was measured by DCFH-DA fluorescent probes (Scale bar=50 *μ*m; (**d** and **e**)) The data are represented as mean±S.E.M. from four experiments. *P<0.05 *versus* the sham group. Twenty-four hours after NMDA injection in the mouse cortex, lesion volume was measured by Nissl staining (Scale bar=500 *μ*m, Upper; 50 *μ*m, Lower; (**f**)) The lower panel in (**f**) is a higher power image of the boxed section in the upper panel, and it shows the loss of neurons and disrupted integrity of the cortex. Neurological deficits were measured by the neurological severity score (NSS; sham group, *n*=6; NMDA group, *n*=9; (**h**)) The serum NSE concentration was also measured ((**i**); the sham group, *n*=7; the NMDA group, *n*=9) 12 and 24 h after injury. Data are represented as mean±S.E.M. *P<0.05 *versus* the sham group

In accordance with the methods reported by Koh and Choi,^26^ we replicated an *in vitro* model of NMDA injury in our experiments. After NMDA injury, lactate dehydrogenase (LDH) concentrations increased when compared with the sham group after injury (sham *versus* NMDA injury: 258.12±9.80 *versus* 368.85±27.56 at 6 h after injury, 289.3±12.38 *versus* 868.6±31.13 at 12 h after injury and 335.4±17.11 *versus* 957.4±27.12 at 24 h after injury; Figure 1a). Furthermore, the apoptotic rate (Hoechst staining; sham *versus* NMDA injury: 4.2±0.18% *versus* 34.1±4.50% Figures 1b and c) and ROS fluorescence intensity (DCFH-DA fluorescence detection; sham *versus* NMDA injury: 1.0±0.01 *versus* 1.90±0.16-fold; Figures 1d and e) increased by when compared with that of the sham group, respectively.

We established a mouse model of NMDA cortical injury that was based on a previous striatum study.^27^ Disrupted cortical integrity and extensive neuronal cell loss on the side ipsilateral to the NMDA injection site were detected by Nissl staining, but no changes were observed on the contralateral side 24 h after injury (Figure 1f). Furthermore, when compared with the sham group, the neurological deficits assessed by the neurological severity score (NSS) were significantly aggravated in the NMDA injury group (sham *versus* NMDA injury: 0.50±0.22 *versus* 14±1.47 points at 12 h after injury, and 0.00±0.00 *versus* 8±1.03 points at 24 h after injury; Figure 1h). Moreover, when compared with the sham group (1.10±0.03 ng/l), significantly increased serum neuron-specific enolase (NSE) levels were observed in the NMDA injury group (12 h after injury: 3.05±0.26 ng/l; 24 h after injury: 4.55±0.31 ng/l; Figure 1i). Thus, we successfully replicated an NMDA injury model *in vitro* and established an NMDA cortical injury model *in vivo*.

### NMDA injury elevated Homer1a expression *in vivo* and *in vitro*

**Figure 2:**
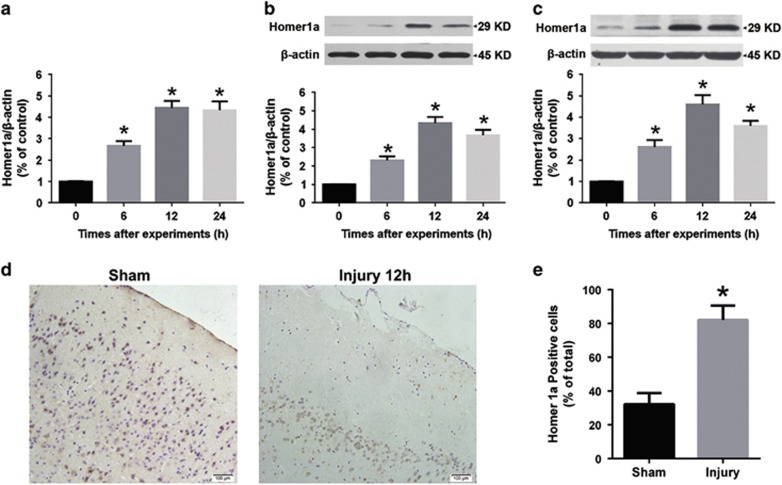
NMDA injury induced Homer1a expression *in vitro* and *in vivo*. After NMDA injury in cultured cortical neurons, Homer1a mRNA was measured by qRT-PCR at different time points (**a**), and Homer1a protein expression was measured by WB. (**b**) Data are represented as mean±S.E.M. from five experiments. *P<0.05 *versus* 0 h group. After NMDA injection in the mouse cortex, Homer1a expression was measured by WB analysis at 6, 12 and 24 h (**c**), *P<0.05 *versus* 0 h (sham) group. Immunohistochemistry staining for Homer1a in the cortex surrounding the lesion area (**d**) and Homer1a-positive neurons were counted 12 h after NMDA injection (**e**; the sham group, *n*=6; NMDA group, *n*=9). Scale bar=100 *μ*m. Data are represented as mean±S.E.M., *P<0.05 *versus* the sham group

To investigate Homer1a expression after NMDA injury, we performed qRT-PCR and western blot (WB) analysis. After NMDA injury *in vitro,* Homer1a mRNA levels increased (6 h, 2.66±0.23; 12 h, 4.44±0.33; and 24 h, 4.33±0.42-fold) when compared with the sham group (1.00±0.01-fold; Figure 2a). Protein expression was also significantly elevated (6 h, 2.31±0.22; 12 h, 4.33±0.34; and 24 h 3.67±0.30-fold), when compared with the sham group (1.00±0.02-fold; Figure 2b). The expression of Homer1a protein was also significantly elevated in the *in vivo* model of NMDA cortical injury (6 h, 2.61±0.32; 12 h, 4.60±0.45; and 24 h, 3.60±0.23-fold) when compared with sham group (1.00±0.01-fold; Figure 2c). Furthermore, the percentage of Homer1a-positive neuronal cells surrounding the lesion in the cortex of the NMDA injury group (32.23±6.56%) was higher than in the sham group (82.12±8.44%) at 24 h (Figures 2d and e). These results indicated that Homer1a expression is upregulated by NMDA injury, and that Homer1a might have a role in NMDA injury.

### Homer1a protected neurons from NMDA injury *in vitro*

**Figure 3:**
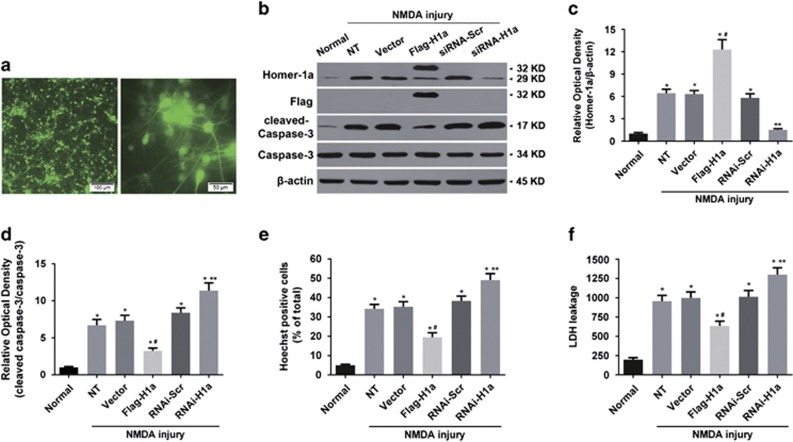
Homer1a protected neurons from NMDA injury *in vitro*. Cultured cortical neurons were infected by LVs carrying pGC-FU-EGFP-3FLAG vectors. A representative fluorescence image (Scale bar=100 *μ*m, Left; 50 *μ*m, Right; **a**). Homer1a expression was assessed by WB in cultured cortical neurons expressing different LVs. (**b** and **c**) After transfection and NMDA injury, caspase 3 cleavage was measured by WB (**b** and **d**), apoptotic rate was determined by Hoechst staining (**e**) and cytotoxicity was assessed by LDH assay (**f**). Data are represented as the mean±S.E.M. from five experiments. *P<0.05 *versus* the normal group; ^#^*P*<0.05 *versus* the non-infection treatment (NT) and vector group; **P<0.05 *versus* the RNAi-Scr group

To study the role of NMDA injury-induced Homer1a in neuronal survival, we altered Homer1a expression using a lentivirus (LV) expression vector. After 3 days of LV-mediated EGFP infection, the rate of EGFP-positive cells was approximately 92% (Figure 3a), indicating that LV infection was highly efficient. Twenty-four hours after injury, Homer1a protein expression increased in the LV-expressed Flag-Homer1a group (Flag-H1a; 12.30±1.32-fold) when compared with the non-transfected group (NT; 6.43±0.56-fold) or the empty vector group (Vector; 6.31±0.49-fold; Figures 3b and c). Furthermore, when compared with NT group, Homer1a overexpression inhibited caspase 3 cleavage (NT *versus* Flag-H1a: 6.71±0.78 *versus* 3.24±0.38-fold; Figures 3b and d), attenuated the rate of apoptosis (NT *versus* Flag-H1a: 34.2±2.26% *versus* 19.5±2.32% Figure 3e) and reduced cytotoxicity (LDH release; NT *versus* Flag-H1a: 957.45±73.17 *versus* 635.20±62.10 ng/l; Figure 3f) in NMDA-injured neurons.

To clarify the effects of endogenous Homer1a on NMDA injury, Homer1a expression was significantly downregulated in LV-expressed Homer1a targeting shRNA group (RNAi-H1a; 1.50±0.17) when compared with the NT group (6.43±0.56-fold; Figures 3b and c). Homer1a downregulation increased caspase 3 cleavage (NT *versus* RNAi-H1a: 6.71±0.78 *versus* 11.37±1.05-fold; Figures 3b and d), the rate of apoptosis (NT *versus* RNAi-H1a: 34.2±2.26% *versus* 49.1±3.35% Figure 3e) and cytotoxicity (NT *versus* RNAi-H1a: 957.45±73.17 ng/l *versus* 1300.4±88.10 ng/l; Figure 3f) in neurons undergoing NMDA injury when compared with the NT group (Figure 3f). These results indicated that downregulation of Homer1a aggravated the neuronal injury induced by NMDA (Figures 3d–f). Above all, these results indicated that Homer1a was an endogenous neuroprotective protein in NMDA injury.

### Homer1a decreased NMDA-induced Ca^2+^ influx, oxidative stress and downstream signaling *in vitro*

**Figure 4:**
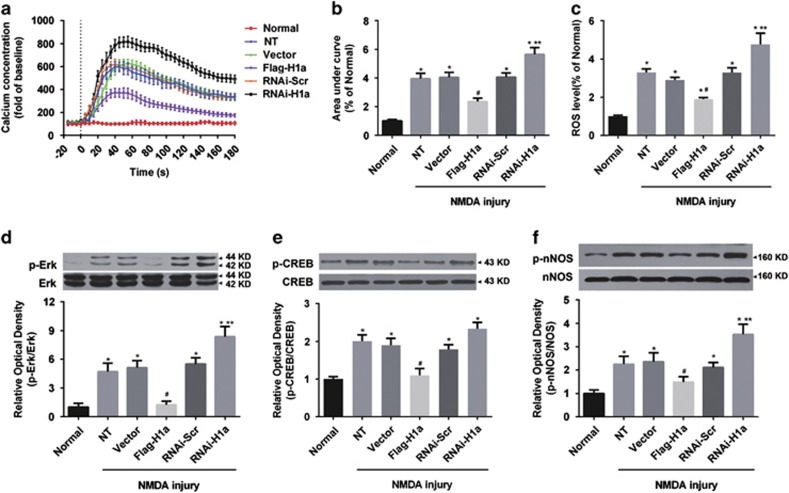
Homer1a decreased NMDA-induced Ca^2+^ influx, oxidative stress and downstream effects *in vitro*. After infection with different LVs, cultured cortical neurons were stimulated with NMDA. Intracellular Ca^2+^ concentrations were measured for up to 30 min (**a**) and the area under the curves was calculated (**b**). ROS production was measured by DCFH-DA fluorescent probe (**c**) 24 h after transfection with different LVs and NMDA injury, the phosphorylation levels of NOS (**d**), ERK (**e**) and CREB (**f**) were measured by WB. Data were represented as mean±S.E.M. from four experiments. *P<0.05 *versus* normal group; ^#^*P*<0.05 *versus* NT and Vector group; **P<0.05 *versus* RNAi-Scr group

After the protective effects of Homer1a in NMDA injury were confirmed, we further examined the effects of Homer1a on Ca^2+^ influx, oxidative stress and downstream signaling pathways induced by NMDA injury. Our results revealed that overexpression of Homer1a significantly alleviated the Ca^2+^ influx (NT *versus* Flag-H1a: 3.96±0.38 *versus* 2.36±0.24-fold; Figures 4a and b) and increased the ROS level (NT *versus* Flag-H1a: 3.30±0.19 *versus* 1.90±0.08-fold; Figure 4c), compared with NT group. RNAi-H1a downregulation of Homer1a had the opposite effect, with significantly increased calcium influx (NT *versus* RNAi-H1a: 3.96±0.38 *versus* 5.65±0.49-fold; Figures 4a and b) and ROS levels (NT *versus* RNAi-H1a: 3.30±0.19 *versus* 4.77±0.57-fold; Figure 4c) when compared with the NT group.

Furthermore, NMDA treatment increased the phosphorylation levels of the resulting downstream signaling molecules, including Erk, CREB and nNOS. Homer1a overexpression significantly decreased p-Erk (NT *versus* Flag-H1a: 4.71±0.88 *versus* 1.24±0.38-fold; Figure 4d), p-CREB (NT *versus* Flag-H1a: 2.01±0.17 *versus* 1.10±0.18-fold; Figure 4e) and p-nNOS (NT *versus* Flag-H1a: 2.25±0.17 *versus* 1.48±0.23-fold; Figure 4f) levels when compared with the NT group. Homer1a downregulation significantly increased p-Erk (NT *versus* RNAi-H1a: 4.71±0.88 *versus* 8.37±1.05-fold; Figure 4d) and p-nNOS (NT *versus* Flag-H1a: 2.25±0.17 *versus* 3.53±0.22-fold; Figure 4f) expression when compared with the un-treated group. Homer1a downregulation had no effect on p-CREB expression (Figure 4e). These data suggest that Homer1a changes the properties of NMDARs, reduces permeability and decreases the activity of NMDAR downstream pathways.

### Homer1a decreased the peak value of NMDAR currents in neurons but had no direct effects on NMDAR currents in HEK293T cells

**Figure 5:**
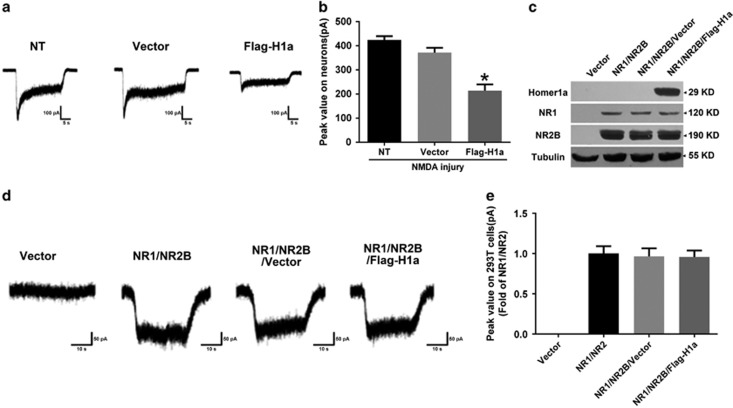
The effect of Homer1a on the peak value of NMDAR currents in neurons and HEK293T cells. After LV-Flag-H1a infection of cultured cortical neurons, whole-cell patch clamp recordings were used to measure NMDAR currents, illustrated by a representative current image (**a**) and the bar graph of peak values (**b**). Expression of Homer1a, NR1 and NR2B in HEK293 cells transfected with NR1/NR2B receptors without (the control) or with Flag-H1a were detected by WB (**c**). Whole-cell patch clamp recordings were used to measure NMDAR currents in co-expressed HEK293 cells, illustrated by a representative current image (**d**) and the bar graph of peak values (**e**). Data were represented as mean±S.E.M. obtained from five experiments.*P<0.05 *versus* the NT group

The above results confirmed that Homer1a protected neurons from NMDA injury, reduced calcium influx, decreased ROS generation and moderated the activity of related downstream signaling pathways. However, the specific mechanisms remained unclear. We speculated that Homer1a activity might rely on NMDAR activity regulation, and, thus, whole-cell recordings of NMDA (100 *μ*M)-evoked partially desensitizing inward currents (INMDA) were examined. Homer1a overexpression decreased the peak amplitude to 50.47% of the NT group (NT *versus* Flag-H1a: 424.22±15.66 *versus* 212.94±29.22 pA; Figures 5a and b). This indicated that Homer1a overexpression reduces NMDAR channel activity.

We next examined whether Homer1a overexpression directly regulated NMDAR channel activity, and whether the NR2B subunits were involved in these regulatory effects in non-neuronal cells. After successful co-expression of recombinant NR1/NR2B receptors with Flag-H1a in HEK293 cells (Figure 5c), we examined the current changes with the recombinant NR1/NR2B receptor. No significant differences were observed in the peak amplitude between the NR1/NR2B/Flag-H1a group and the NR1/NR2B or NR1/NR2B/vector groups (Figures 5d and e). These results suggested that Homer1a changes NMDAR properties in neurons, but that it might not directly regulate NMDAR channel activity.

### Homer1a reduces the membrane distribution of NMDAR in cultured cortical neurons

**Figure 6:**
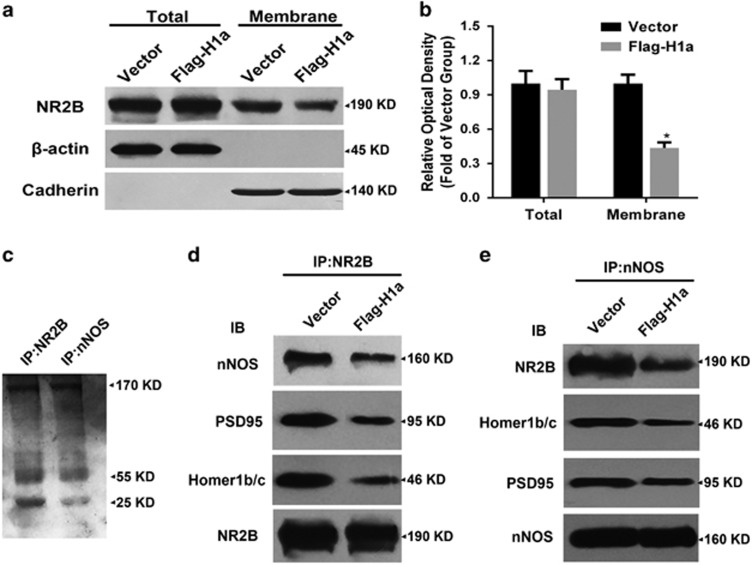
Effects of Homer1a on the distribution of NR2B and NR2B-PSD95-nNOS complexes. After infection with LV-Flag-H1a and NMDA injury, NR2B levels in total protein and membrane protein extracts was measured by WB (**a**). NR2B levels in total protein was standardized to β-actin and NR2B levels in membrane extracts was standardized to cadherin (**b**). After infection with LV-Flag-H1a and NMDA injury in cortical neurons, the immuneprecipitate with antibodies against NR2B and nNOS in the extracts from cultured cortical neurons was stained with coomassie blue (**c**). Extracts from cultured cortical neurons transfected with LV-Flag-H1a or vector and subjected to NMDA injury were precipitated with antibodies against NR2B (**d**) or nNOS (**e**) and probed with antibodies against nNOS, PSD95, Homer1b/c and NR2B. Data were represented as mean±S.E.M. obtained from four experiments. *P<0.05 *versus* the Vector group

NMDAR channel activity and distribution are critical factors in NMDAR function.^28, 29^ We observed that Homer1a changed NMDAR properties, but did not directly regulate channel activity; therefore, we speculated that Homer1a might regulate NMDAR distribution. To test this hypothesis, neurons were infected with LV-Flag-H1a, and then neuron total protein and membrane protein fractions were isolated to investigate the distribution of the NMDAR subunit, NR2B. Homer1a overexpression had no effect on total NR2B levels, but significantly decreased the membrane distribution of NR2B to 50.23% when compared with the vector group (Vector *versus* Flag-H1a: 1.00±0.08 *versus* 0.43±0.05-fold; Figures 6a and b). This suggested that Homer1a might decrease the membrane distribution of NMDARs, which might contribute to Homer1a's NMDA injury protective effects.

### Homer1a disrupted the physical interaction of NR2B, PSD95 and nNOS in cultured cortical neurons

After our observations confirming Homer1a regulation of NR2B distribution, we explored the regulatory mechanisms underlying this phenomenon. We used anti-NR2B antibody and anti-nNOS antibody to precipitate NMDAR complexes from cultured cortical neurons to study the effects of Homer1a on NR2B-PSD95-nNOS complexes. Precipitates were separated by SDS-PAGE and stained with coomassie blue (Figure 6c). Examining NR2B immunoprecipitations, we found that Flag-Homer1a upregulation reduced the NR2B-bound nNOS, PSD95 and Homer1b/c when compared with the empty vector group (Vector *versus* Flag-H1a: nNOS, 1.00±0.14 *versus* 0.51±0.10-fold; PSD95, 1.00±0.11 *versus* 0.47±0.16-fold; Homer1b/c, 1.00±0.07 *versus* 0.38±0.09-fold; Figure 6d). We also carried out the reciprocal co-immunoprecipitation, in which we observed decreased NR2B, Homer1b/c and PSD95 in precipitated nNOS complexes when compared with the vector group (Vector *versus* Flag-H1a: NR2B, 1.00±0.09 *versus* 0.40±0.13-fold; Homer1b/c, 1.00±0.12 *versus* 0.47±0.09-fold; PSD95, 1.00±0.07 *versus* 0.60±0.09-fold; Figure 6e). These results suggested that Homer1a could disrupt the NR2B-PSD95-nNOS complex by competitive binding with Homer1b/c. This may be the critical mechanism underlying the protective effects of Homer1a against NMDA injury.

### Homer1a reduced brain damage and improved neurological function after NMDA injury *in vivo*

**Figure 7:**
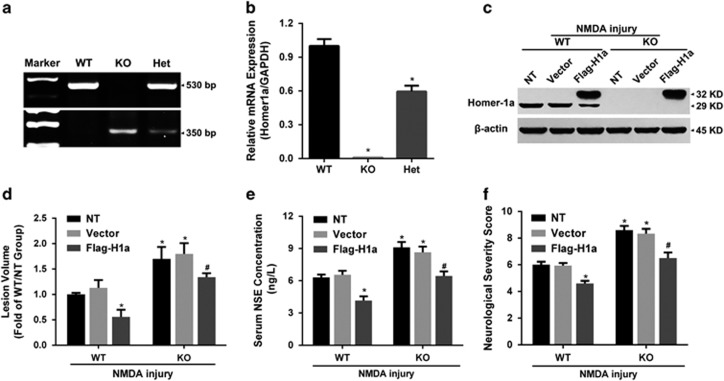
Homer1a reduced brain damage and improved neurological function after NMDA injury *in vivo*. Homer1a KO mice were confirmed and identified with DNA PCR (**a**) and qRT-PCR (**b**) analysis. After 7 days of stereotaxic injections of LV-Flag-H1a in Homer1a WT and KO mice, *in vivo* NMDA injury was induced. Homer1a was detected by WB (**c**), lesion volume was measured by Nissl staining (**d**), serum NSE levels (**e**) were measured and neurological deficits were evaluated by NSS (**f**; the NT group, WT *n*=8, KO *n*=6; the Vector group, WT *n*=7, KO *n*=6; the Flag-H1a group, WT *n*=7, KO *n*=6). Data are represented as mean±S.E.M. *P<0.05 *versus* the WT-NMDA injury and WT-Vector-NMDA injury groups; ^#^*P*<0.05 *versus* the KO-NMDA injury, the KO-Vector-NMDA injury and the WT-Flag-H1a-NMDA injury groups

Our results indicated that Homer1a protected neurons from NMDA injury by disrupting NR2B-PSD95-nNOS complexes and reducing NMDAR membrane distribution *in vitro*. To test whether Homer1a was protective *in vivo*, we subjected Homer1a KO mice to NMDA injury. Homer1a KO mice were confirmed by DNA PCR (Figure 7a) and qRT-PCR (Figure 7b), which showed that Home1a mRNA was absent in KO mice and reduced in heterozygous mice. Twenty-four hours after NMDA injury, Homer1a KO mice had more severe neuronal injuries when compared with Homer1a WT mice. Exacerbated injuries included: enlarged lesion volume (WT-NT-NMDA injury *versus* KO-NT-NMDA injury: 1.00±0.03 *versus* 1.7±0.24-fold; Figure 7d), increased serum NSE levels (WT-NT-NMDA injury *versus* KO-NT-NMDA injury: 6.31±0.27 *versus* 9.10±0.55 ng/l; Figure 7e) and reduced neurological function (WT-NT-NMDA injury *versus* KO-NT-NMDA injury: 6.00±0.25 *versus* 8.6±0.25 points; Figure 7f). These results demonstrated that the *Homer1a* knockout mice were more susceptible to NMDA-induced brain injury, indicating that Homer1a might have protective effects against NMDA injury *in vivo*.

To further examine the protective effects of Homer1a overexpression in NMDA injury in mice, we infused LV-Flag-H1a into the right cortex of WT and Homer1a KO mice. Seven days after LV injection, mice were subjected to NMDA injury. Successful Homer1a overexpression was confirmed by WB (Figure 7c). In Homer1a WT mice, Homer1a overexpression in the NMDA-injured hemisphere protected from NMDA injury. Homer1a-overexpressing mice had reduced lesion volume (WT-NT-NMDA injury *versus* WT-Flag-H1a-NMDA injury: 1.00±0.05 *versus* 0.51±0.15-fold; Figure 7d), decreased serum NSE levels (WT-NT-NMDA injury *versus* WT-Flag-H1a-NMDA injury: 6.31±0.27 *versus* 4.15±0.43 ng/l; Figure 7e) and improved neurological function (WT-NT-NMDA injury *versus* WT-Flag-H1a-NMDA injury: 6.00±0.25 *versus* 4.60±0.20-points; Figure 7f). No significant differences were observed between the NT-NMDA injury group and the Vector-NMDA injury group in WT and KO mice. Notably, Homer1a overexpression in KO mice resulted in similar outcomes when the KO-NT-NMDA injury and KO-Vector-NMDA injury groups were compared (Figures 7d–f). However, a significant increase in neuronal injury severity was observed in the KO-Flag-H1a-NMDA injury group when compared with the WT-Flag-H1a-NMDA injury group. The KO-Flag-H1a-NMDA injury group had enlarged lesion volume (WT-Flag-H1a-NMDA injury *versus* KO-Flag-H1a-NMDA injury: 0.51±0.15 *versus* 1.39±0.21-fold; Figure 7d), increased serum NSE levels (WT-Flag-H1a-NMDA injury *versus* KO-Flag-H1a-NMDA injury: 4.15±0.43 *versus* 6.45± 0.42 ng/l; Figure 7e) and improved neurological function (WT-Flag-H1a-NMDA injury *versus* KO-Flag-H1a-NMDA injury: 4.60±0.20 *versus* 6.50±0.50 -points; Figure 7f). Thus, we confirmed that Homer1a overexpression decreased NMDA-induced brain injury *in vivo*.

## Discussion

We demonstrated that Homer1a conferred neuroprotection against NMDA-induced neuronal injury. The potential underlying mechanisms of this neuroprotection that we observed included the following: Homer1a disrupted NMDAR-PSD95-nNOS complexes by competitively replacing Homer1b/c; The Homer1a weakened stability of the NMDAR complexes and decreased the membrane distribution of NMDARs, resulting in reduced NMDAR activity; NMDAR-induced Ca^2+^ influx, ROS generation and activation of downstream signaling pathways were significantly inhibited by Homer1a, and, thus, NMDA-induced neuronal injury was alleviated and neurological function was improved.

NMDARs have well-established roles in traumatic brain injury and cerebral ischemia,^30, 31^ and a number of inhibitors targeting NMDAR-PSD95-nNOS complexes have been thoroughly investigated.^32, 33, 34, 35, 36, 37^ In the present study, we successfully established *in vivo* and *in vitro* NMDA injury models. Consistent with previous studies, we confirmed that NMDA treatment induced a large Ca^2+^ influx, ROS generation and activation of downstream signaling pathways, all of which were related to NMDA-induced neuronal injury.^38^ Homer1a regulation of calcium homeostasis and receptor trafficking has been reported in several neurological diseases.^39, 40^ Our previous study found that Homer1a protected against traumatic brain injury by regulating group I mGluRs.^41^ In this study, we observed Homer1a protected neurons from NMDA-induced neuronal injury and apoptosis. It has been suggested that Homer1a functions in neuroprotection after brain injury by regulating both mGluRs and ionic glutamate receptors.

As discussed above, NMDA injury-induced Ca^2+^ influx, ROS generation and activation of downstream signaling pathways contributed to neuronal injury. Previous studies have indicated that Homer1a exerts neuroprotection by regulating mGluRs, inhibiting Ca^2+^ overload^42, 43^ and alleviating oxidative stress.^41, 44^ Therefore, It is unclear whether Homer1a exerted neuroprotection in NMDA injury by affecting Ca^2+^ influx or not. Our present study found that Homer1a upregulation decreased Ca^2+^ influx, whereas Homer1a downregulation had the opposite effect. Additionally, NMDAR-induced Ca^2+^ influx, induced by NMDAR activation, could cause enzyme overstimulation (such as calpains, protein kinases, nNOS, calcineurins and endonucleases). This enzyme overstimulation induced oxidative stress and mitochondrial dysfunction, leading to apoptosis.^7, 45, 46^ Consistent with previous studies,^41, 47, 48^ Erk, CREB and nNOS activation were observed after NMDA injury in our study. Furthermore, a reduction in oxidative stress and reduced Erk, CREB and nNOS activation was observed after Homer1a overexpression, suggesting that Homer1a has a critical role in regulating NMDAR function and downstream signaling.

Several inhibitors (including Ro25-6981, Tat-NR2B9c, Tat-NPEG4 and ZL-006) that target NMDARs or NMDAR-PSD95-nNOS complexes have shown neuroprotective abilities that rely on reducing NMDA-induced Ca^2+^ influx or nNOS activation.^32, 37, 49, 50^ Our investigation indicated that Homer1a functionally regulated NMDAR channel activity in neurons by showing that Homer1a expression decreased the peak value of NMDARs currents. Interestingly, Homer1a overexpression did not directly regulate recombinant NR1/NR2B receptor currents in non-neuronal cells, such as HEK293 cells. One possible explanation for this discrepancy is that there are more synaptic scaffolding proteins maintaining the stability of NMDARs in neurons when compared with HEK293 cells. Therefore, Homer1a might regulate NMDARs by targeting these scaffolding proteins.

It has been shown that NMDAR membrane distribution was altered in experimental models of ischemia and Parkinson's disease.^28, 32, 51^ Our study found that Homer1a overexpression significantly decreased the membrane distribution of NMDARs after NMDA injury, which might have a close correlation with the observed decrease in Ca^2+^ influx and NMDAR currents. Previous studies have demonstrated that the membrane distribution of NMDAR is affected by both membrane targeting and cycling of NMDAR.^52^ NMDAR targeting is primarily regulated by synaptic scaffolding proteins, including PSD95 and synapse-associated protein 102.^53^ Additionally, Homer1b/c facilitates synaptic scaffolding protein trafficking, is extensively involved in mGluRs signaling and is competed by Homer1a. Therefore, we speculated that Homer1a might negatively affect trafficking, and then disrupt membrane targeting.

NMDAR endocytosis and insertion into the membrane are two important aspects of NMDAR cycling.^52^ NMDAR endocytosis is a fine-tuning procedure that moderates various physiological and pathophysiological activities.^52^ For example, increasing NMDAR endocytosis reduces excitotoxic vulnerability to NMDA in dopaminergic neurons.^28^ Synaptic scaffolding proteins such as Shank, PSD95 and Homer1b/c affect NMDAR endocytosis by influencing the stability of NMDAR complexes. Homer1b/c contributes to NMDA-PSD95-Shank-Homer1b/c complex formation and regulates the activity of NMDAR in neurons.^12, 20^ Therefore, we speculated that Homer1a might negatively modulate the stability of NMDAR complexes by acting as a Homer1b/c competitor. This was evidenced by our results demonstrating that Homer1a overexpression weakened the physical associations of NR2B, PSD95, Homer1b/c and nNOS. Additionally, protein kinase C promoted NMDAR delivery and insertion into neuronal membranes.^54, 55^ Our previous study found that Homer1a overexpression inhibited protein kinase C activity in the traumatic brain injury model.^41^ Therefore, Homer1a might inhibit or reduce NMDAR delivery and insertion by regulating protein kinase C activity. In conclusion, our results suggest that Homer1a protects neurons from NMDA injury by reducing the membrane distribution of NMDAR, and this might owe to poor NMDAR plasma membrane targeting and decreased NMDAR cycling. However, the exact mechanisms underlying this process still need to be clarified by future studies.

We also demonstrated that Homer1a KO mice had more severe neuronal injury and increased neurological deficits when compared with WT mice. This implied that the loss of the *Homer1a* gene was related to increased NMDA-induced brain injury severity. More importantly, the neuroprotective function of Homer1a was supported by the fact that Homer1a overexpression alleviated NMDA-induced brain injuries. This benefit was seen equally in both Homer1a WT and KO mice. This leads to the conclusion that Homer1a could be a therapeutic target in diseases related to NMDA injury. Although the intra-cortical LV injections in the present study were used mainly as proof-of-principle tools for the verification of Homer1a efficacy, as previously described,^41^ our current results represent the foundation of future development of clinical applications of Homer1a with less invasive tools. Additionally, considering the number of alternate factors that can potentially be used to upregulate Homer1a expression, such as BDNF^56^ and pituitary adenylate cyclase-activating polypeptide,^57^ there is a good chance that therapeutic expression of Homer1a will be investigated and successfully developed in future clinical studies.

In conclusion, we demonstrated that Homer1a protected against NMDA-induced neuronal injury by disrupting NR2B-PSD95-nNOS complexes and reducing the membrane distribution of NMDARs. Future studies are needed to further investigate the mechanisms of neuroprotection in NMDA injury, Homer1a targeting and the long-term effects of Homer1a expression.

## Materials and Methods

### Animals

Male C57BL/6 J mice (10–12-weeks old) were obtained from the Laboratory Animal Center of the Fourth Military Medical University. Mice were given free access to food and water and were housed in standard cages in a room maintained at 20–22°C with a 12 h light/dark cycle. All experimental protocols and animal handling procedures were performed in accordance with the National Institutes of Health Guidelines on the Use of Laboratory Animals, and were approved by the Institutional Animal Care and Use Committee of the Fourth Military Medical University.

Homer1a KO mice were kindly provided by Paul Worley.^42, 58^ We crossed heterozygous Homer1a mice (Het, Homer1a^+/−^) to generate wild-type (WT, Homer1a^+/+^) and knockout (KO, Homer1a^−/−^) mice. Ten- to 12-week-old males were prepared and treated as described below.

### Cortical neuronal culture

Cortical neurons were cultured from C57BL/6 J mice using a method modified from the protocol reported by Redmond *et al.*^59^ Briefly, cerebral cortices were removed from 16–18-day-old embryos, stripped of meninges and blood vessels and minced. Tissues were dissociated by 0.25% trypsin digestion and gentle trituration. Neurons were re-suspended in Neurobasal medium containing 2% B27 supplement and 0.5 mM L-glutamine and plated at a density of 3 × 10^5^ cells/cm^2^. Petri dishes were coated with poly-D-lysine at room temperature (RT) overnight before seeding. Cultures were maintained at 37°C in a humidified 5% CO_2_ incubator. Cultured neurons were verified at >95% by β-III-tubulin and GFAP staining and used for *in vitro* studies on days 15 through 18.

### Cortex microinjection of NMDA and measurement of lesion volume

The *in vivo* NMDA injury model was created using a stereotaxic cortical injection as previously described in the striatal brain^5, 27^ with some modifications. NMDA (Sigma-Aldrich, St. Louis, MO, USA) was dissolved at a final concentration of 50 mM in Tris-buffered control salt solution.^26^ Mice were anesthetized with isoflurane (3% for induction and 1.5% for maintenance) and the head was fixed to a stereotaxic frame. The cortical injection of the NMDA solution was performed in the right hemisphere (anterior to the bregma, 0.5 mm; lateral to the bregma, 2.5 mm; ventral to the bregma, 1.8 mm) using a microinjection system (volume: 1 *μ*l, speed: 0.25 *μ*l/min, the needle was left *in situ* for an additional 10 min). The injection procedure in the sham-control groups was performed in a manner similar to the NMDA injury group, but without injection of the NMDA solution. After injections, mice were placed in a 37°C chamber, and then returned to their cages after full recovery from anesthesia. To study lesion development, mice were decapitated 24 h after NMDA injection under deep anesthesia with 100 mg/kg pentobarbital. Brain tissues were rapidly dissected, fixed in 4% formalin and embedded in paraffin. Serial 20*-μ*m-thick coronal sections were taken and every tenth section was stained with cresyl violet as previously described.^60^ The lesion area was identified by the loss of Nissl bodies, measured by image analysis and integrated to calculate the lesion volume.^5^

### *In vitro* model of NMDA injury

Cortical neurons were exposed to NMDA according to the method reported by Koh and Choi.^26^ NMDA was dissolved at a final concentration of 300 mM in control salt solution. Cultured neurons were washed three times with control salt solution, and then exposed to NMDA for 10 min. Next, the exposure solution was washed away and replaced by neurobasal medium. Finally, the cells were placed in an incubator for 20–24 h prior to subsequent experiments.

### Western blotting

WB analysis was performed as previously described.^61^ After various treatments, proteins were extracted, quantified and separated by electrophoresis. After electrophoresis, the proteins were transferred to nitrocellulose membranes and blocked with 5% skim milk. After blocking, the membranes were incubated with the appropriate primary antibodies: goat anti-Homer1a (1 : 200; Santa Cruz, Dallas, TX, USA), mouse anti-Homer1b/c (1 : 1000; Santa Cruz), rabbit anti-PSD95 (1 : 1000; Santa Cruz), rabbit anti-p-Erk, Erk, NR1, NR2B, p-nNOS, nNOS, CREB, p-CREB (1 : 1000; Cell Signaling Technology (CST), Danvers, MA, USA) and mouse anti-*β*-Actin (1 : 2000; Sigma-Aldrich). Immunoreactivity was detected by incubation with horseradish peroxidase-conjugated horse anti-secondary antibodies (1 : 20000; CST) followed by chemiluminescent substrate development (Thermo Fisher Scientific Inc., Waltham, MA, USA). WBs for each protein were repeated three times and the optical densities of the bands were calculated using a MiVnt image analysis system (Bio-Rad Laboratories, Inc., Hercules, CA, USA).

### Quantitative reverse transcription-PCR (qRT-PCR)

RNA was isolated from cultured cortical neurons using Trizol (Invitrogen, Life Technologies, Grand Island, NY, USA). Two micrograms of template RNA were used for first strand cDNA synthesis using a reverse transcription kit (Takara, Dalian, China). qRT-PCR was performed with CFX-96 (Bio-Rad). Amplified product specificity was confirmed by the examination of dissociation plots and gel electrophoresis. The following primers were used for real-time PCR:

Homer1a primers:

5′- GGC AAA CAC TGT TTA TGG ACT GG - 3′ (forward)

5′- GTA ATT CAG TCA ACT TGA GCA ACC - 3′ (reverse)

β-actin primers:

5′- CTA AGG CCA ACC GTG AAA AGA TG - 3′ (forward)

5′- ACC GCT CGT TGC CAA TAG TGA TG - 3′ (reverse)

qPCR was conducted according to the following conditions: 94°C (30 s), 58°C (30 s) and 72°C (30 s) for 50 cycles. Samples were tested in triplicate and data from five independent experiments were used for analysis. Relative gene expression was calculated using the 2^−ΔΔCT^ method.

### Immunohistochemistry

Twenty-four hours after injury, animals were humanely killed and transcardially perfused with 4% paraformaldehyde. Brain tissues were embedded in paraffin, sectioned and deparaffinized prior to analysis. The avidin–biotin–peroxidase complex (ABC) method was used in this study. After inactivation of endogenous peroxide with 0.3% H_2_O_2_, deparaffinized sections were permeabilized with 0.2% Triton X-100 and blocked with donkey serum. After blocking, sections were incubated at 4°C overnight with the Home1a antibody (1 : 50, Santa Cruz). The next day, sections were incubated with biotinylated immunoglobulin G antibodies (Sigma-Aldrich) for 2 h at RT and then incubated with ABC kit reagents (Sigma-Aldrich) for 2 h at RT. The sections were developed in a staining solution containing 3, 3′-diaminobenzidine for 5–10 min at RT. Sections were examined under a light microscope for satisfactorily developed staining.

### Hoechst staining

Neuronal death was visualized with Hoechst 33342 staining. Twenty-four hours after injury, neurons were fixed for 20 min with 4% paraformaldehyde at RT and then stained for 15 min with 1 mg/ml Hoechst 33342 (Sigma-Aldrich) in incubation buffer. Apoptosis was observed under a fluorescence microscope. Neurons with fragmented or condensed nuclei were counted and expressed as percentage of the total population. Six different fields were counted per well in six separate experiments.

### Cytotoxicity assay

Cytotoxicity was evaluated by LDH concentration, as previously described.^41^ The supernatant was collected from cell culture plates and the LDH content was determined using an LDH assay kit according to the manufacturer's instructions (Nanjing Institute of Jianchen Biological Engineering, Nanjing, China). LDH levels were evaluated by an automatic biochemical analysis system. LDH cytotoxicity was calculated according to the following formula: LDH cytotoxicity=(sample OD−blank OD)/(standard solution OD−blank standard solution OD) × 2000.

### Neurological severity score (NSS)

The NSS is highly correlated with brain damage severity. Modified NSS was measured based on previously described NSS.^41, 62^ NSS evaluation was conducted by an investigator who was blinded to the experimental groups. The score consists of 10 individual neurological parameters, including motor function, alertness and physiological behaviors. One point is awarded for the lack of a tested reflex or for the inability to perform a task. Thus, the higher score, the more severe is the injury.

### Calcium concentration in neurons

Cytoplasmic Ca^2+^ concentration were determined using the calcium indicator Fura-2AM (Molecular Probes, Life Technologies). Briefly, after being washed with PBS, cortical neurons cultured on coverslips were loaded with Fura-2AM (5 *μ*M) in Hanks Balanced Salt Solution supplemented with 20 mM D-glucose and 10 mM HEPES (HBSS; Gibco, Life Technologies) for 45 min, and then equilibrated for 30 min in the dark at RT. Next, coverslips were tightly mounted on an open-bath imaging chamber containing HBSS. Using a Nikon inverted epifluorescence microscope, neurons were excited at 345 and 385 nm and the emission fluorescence at 510 nm was recorded. Images were collected and analyzed with the MetaFluor image-processing software (Universal Imaging Corp, Downingtown, PA, USA). Ca^2+^ concentration values were calculated and Ca^2+^-insensitive fluorescence was subtracted from each wavelength before calculations.

### Intracellular ROS measurement

Intracellular ROS was monitored using DCFH-DA fluorescent probes (Molecular Probes).^63^ Briefly, neurons were incubated for 30 min with 10 mM DCFH-DA at 37°C, and then washed twice with PBS. Finally, the fluorescence intensity of DCF was measured in a microplate reader with an excitation wavelength of 485 nm and an emission wavelength of 535 nm.

### LV preparation for RNAi and overexpression

The methods for LV preparation of RNAi and overexpression were the same as previously described.^41^ To develop shRNA LVs, a siRNA oligo directed to the coding region+1190 to 1208 or a negative control shRNA (5′- TTC TCC GAA CGT GTC ACG T -3′) was subcloned into a LV expression vector, pFU-GW-RNAi (GeneChem Company, Shanghai, China). The LV construct coexpressed EGFP driven by the ubiquitin C promoter. The U6 promoter drove the shRNA. The LV overexpression system was developed by removing the EGFP open reading frame from the pGC-FU-EGFP-3FLAG construct (GeneChem Company) with an *Age*I/*Nhe*I digestion and replacing it with Homer1a cDNA. Primary cortical neurons were transfected with the LV vectors (MOI=20) for 72 h and then subjected to various subsequent experiments.

### HEK293 cell culture and transient transfection

HEK293 cells were cultured in DMEM supplemented with 10% fetal bovine serum in a 37°C humidified atmosphere with 5% CO_2_. For transfection, cells were seeded into 35-mm petri dishes at 1 × 10^6^ cells/cm^2^. After 24 h, HEK293 cells were transfected with 2 *μ*g pNR1 and 2 *μ*g pNR2B-GFP per well with Lipofectamine 2000 reagent (Invitrogen).^64, 65^ Six hours after transfection, cells were supplemented with fresh culture medium. Forty-eight hours after transfection, HEK293 cells were used for WB and electrophysiological experiments. NR1 and NR2B-GFP plasmids were kindly gifted by Prof. Jian-hong Luo at Zhejiang University School of Medicine.^66^

### Whole-cell patch clamp recordings

Whole-cell recordings were performed at RT on mouse cortical neurons (15–18 days) or HEK293 cells, as previously described.^31, 67^ Cells were transferred to a recording chamber that was continuously perfused with extracellular solution (normal ECS) containing the following (in mM): 140 NaCL, 5 KCL, 2.5 CaCL_2_, 1 MgCL_2_, 10 HEPES and 10 glucose, with pH 7.3 and at a concentration of 310–320 mOsm/l. Patch pipettes were filled with an intracellular solution composed of the following (in mM): 120 K-Gluconate, 5 NaCL, 1 MgCL_2_, 0.2 EGTA, 2 MgATP, 0.1 Na_3_GTP, 10 HEPES and 10 phosphocreatine disodium, with pH 7.2 and at a concentration of 295–310 mOsm/l. Whole-cell recordings were carried out using an Axopatch 200 A amplifier (Molecular Devices, Sunnyvale, CA, USA). Electrode resistances were 3–5 MΩ. During recording, cells were perfused with Mg^2+^-free ECS containing the following (in mM): 140 NaCl, 5 KCl, 2.5 CaCl_2_, 10 HEPES and 10 glucose. Whole-cell NMDA currents were recorded at a holding membrane potential of −60 mV under a voltage-clamped configuration. Currents were evoked by 100 *μ*M NMDA in Mg^2+^-free ECS with 1 *μ*M tritated tetrodotoxin and 10 *μ*M glycine using a Y-tube perfusion system.^68, 69^ Recordings were low-pass filtered at 2 kHz, sampled at 10 kHz and stored as data files using pClamp 10.0 (Molecular Devices). Series resistance was monitored throughout each recording, and if it varied by more than 10%, the recording was rejected.

### Co-immunoprecipitation and membrane protein extraction

For co-immunoprecipitation, neurons were cultured in 10-cm dishes and then harvested in ice-cold lysis buffer containing (in mM): 50 Tris-HCl, 150 NaCL, 1% NP-40, 2 EDTA, 1% Na-orthovanadate (pH 7.4) and proteinase inhibitor mixture (Sigma-Aldrich). After clearing debris by centrifuging at 14 000 × *g* and 4°C, protein concentrations in the extracts were determined using a BCA protein assay kit (Pierce Biotechnology, Thermo Fisher Scientific Inc.). Extracts (800 mg protein) were incubated overnight at 4°C with non-specific IgG (2 mg), poly-clonal rabbit anti-NR2B or anti-nNOS (1:40; CST), followed by the addition of 40 *μ*l of protein G-sepharose (Sigma-Aldrich) for 3 h at 4°C. The precipitates were washed four times with lysis buffer, denatured with SDS sample buffer and prepared for WB. For total protein, after various treatments, petri dishes were washed with ice-cold PBS and lysed with a lysis buffer containing protease inhibitors. For membrane protein extraction, the MEM-PER mammalian membrane protein extraction kit (Pierce) was used according to the manufacturer's instructions. Membrane proteins were washed and concentrated using a Pierce SDS-PAGE Sample Prep Kit (Pierce).

### Stereotaxic injection of LV

LV vector delivery was carried out *in vivo* with stereotaxic cortical injections, as previously described^41^ with some modifications. Three cortical injections were performed in the right hemisphere (ipsilateral to the lesion) as follows: point 1 (anterior to the bregma, 1.0 mm; lateral to the bregma, 2.5 mm; ventral to the bregma, 1.8 mm), point 2 (anterior to the bregma, 0 mm; lateral to the bregma, 2.5 mm; ventral to the bregma, 1.8 mm), point 3 (anterior to the bregma, 0.5 mm; lateral to the bregma, 2.0 mm; ventral to the bregma, 1.8 mm). Each injection contained 1.5 *μ*l of LV suspension (5 × 10^8^ TU/ml), and was delivered at a rate of 0.2 *μ*l/min. The needle was withdrawn after an additional 5 min. After 7 days of LV injection, mice were subjected to NMDA injury as described above.

### Statistical analysis

All experiments were performed at least three times. Values are expressed as mean±S.E.M., and were analyzed by ANOVA followed by Bonferroni's multiple comparisons or unpaired *t*-test with SPSS 22.0 statistical software (IBM Corp., Armonk, NY, USA). *P*<0.05 was considered statistically significant.

## Abbreviations

NMDAR: N-methyl-D-aspartate receptor
nNOS: neuronal nitric oxide synthase
KO: knockout
WT: wild type
Glu: Glutamate
PSD: postsynaptic density
mGluR: metabotropic glutamate receptors
CC: coiled-coil
LDH: lactate dehydrogenase
NSS: neurological severity score
NSE: neuron specific enolase
WB: Western blot
LV: lentivirus
NT: non-transfected
INMDA: NMDA-evoked partially desensitizing inward currents
RT: room temperature
CST: Cell Signaling Technology
qRT-PCR: quantitative reverse transcription-PCR
ABC: avidin–biotin–peroxidase complex
OD: optical density
ECS: extracellular solution
